# Primary pericardial synovial sarcoma: diagnosis of recurrence via multimodal imaging—a case report

**DOI:** 10.1093/ehjcr/ytaf132

**Published:** 2025-03-17

**Authors:** Kanna Nakamura, Taiji Okada, Jiro Esaki, Shigeo Hara, Yutaka Furukawa

**Affiliations:** Department of Cardiovascular Medicine, Kobe City Medical Center General Hospital, 2-1-1 Minatojima-minamimachi, Chuo-ku, Kobe 6500047, Japan; Department of Cardiovascular Medicine, Kobe City Medical Center General Hospital, 2-1-1 Minatojima-minamimachi, Chuo-ku, Kobe 6500047, Japan; Department of Cardiothoracic Surgery, Kobe City Medical Center General Hospital, Kobe 6500047, Japan; Department of Diagnostic Pathology, Kobe City Medical Center General Hospital, Kobe 6500047, Japan; Department of Cardiovascular Medicine, Kobe City Medical Center General Hospital, 2-1-1 Minatojima-minamimachi, Chuo-ku, Kobe 6500047, Japan

**Keywords:** Case report, Synovial sarcoma, Pericardial tumour, Cardiac tumour, Multimodality imaging

## Abstract

**Background:**

Primary pericardial synovial sarcoma (PSS) is an extremely rare malignancy. Diagnosis and management primarily rely on multimodal imaging, with definitive diagnosis typically requiring a biopsy for histopathological confirmation. Although no established treatment protocol exists, complete tumour resection followed by adjuvant chemotherapy and radiotherapy is often considered.

**Case summary:**

A 68-year-old man presented with 1 month of shortness of breath and no significant medical history. Initial examination revealed cardiomegaly, a large pericardial effusion, and a solid mass adjacent to the right atrium. Multimodal imaging, including contrast-enhanced computed tomography and positron emission-T, identified a pericardial mass with metabolic activity. A thoracoscopic biopsy revealed atypical spindle-shaped cells, confirming the diagnosis of synovial sarcoma. This led to total tumour excision and lymph node dissection. Eighteen months post-surgery, imaging revealed recurrence, and the mass from the pericardium near the superior vena cava was resected via thoracotomy. Histopathological examination confirmed recurrent synovial sarcoma. Postoperative chemotherapy was administered; however, lung metastases developed, leading to further treatment 12 months post second surgery.

**Discussion:**

PSS is associated with poor prognosis and a high recurrence risk, highlighting the importance of regular follow-up imaging. While complete surgical resection remains the primary treatment, adjunct therapies may improve outcomes. This case underscores the need for multimodal imaging in diagnosing and monitoring PSS.

**Learning points**

- Primary pericardial synovial sarcoma (PSS) is an extremely rare malignant tumour with no established treatment. Complete surgical resection is the mainstay, but the high risk of recurrence necessitates close follow-up. Adjuvant therapies may be considered to prevent or manage recurrence.
- Multimodal imaging plays a significant role in the diagnosis and monitoring of PSS, which is associated with a poor prognosis and a high risk of recurrence.

## Introduction

Primary pericardial synovial sarcoma (PSS) is an extremely rare malignant tumour with atypical symptoms and presentations.^1^ Diagnosing and managing PSS require multimodal imaging; however, establishing a definitive diagnosis based on imaging alone is challenging. Therefore, histopathological confirmation via biopsy is essential.^2^ Owing to the rarity of this tumour, established treatment protocols are currently lacking. Herein, we report a case of primary PSS, focusing on the diagnostic process and detection of recurrence using multimodal imaging techniques.

## Summary figure

**Figure:**
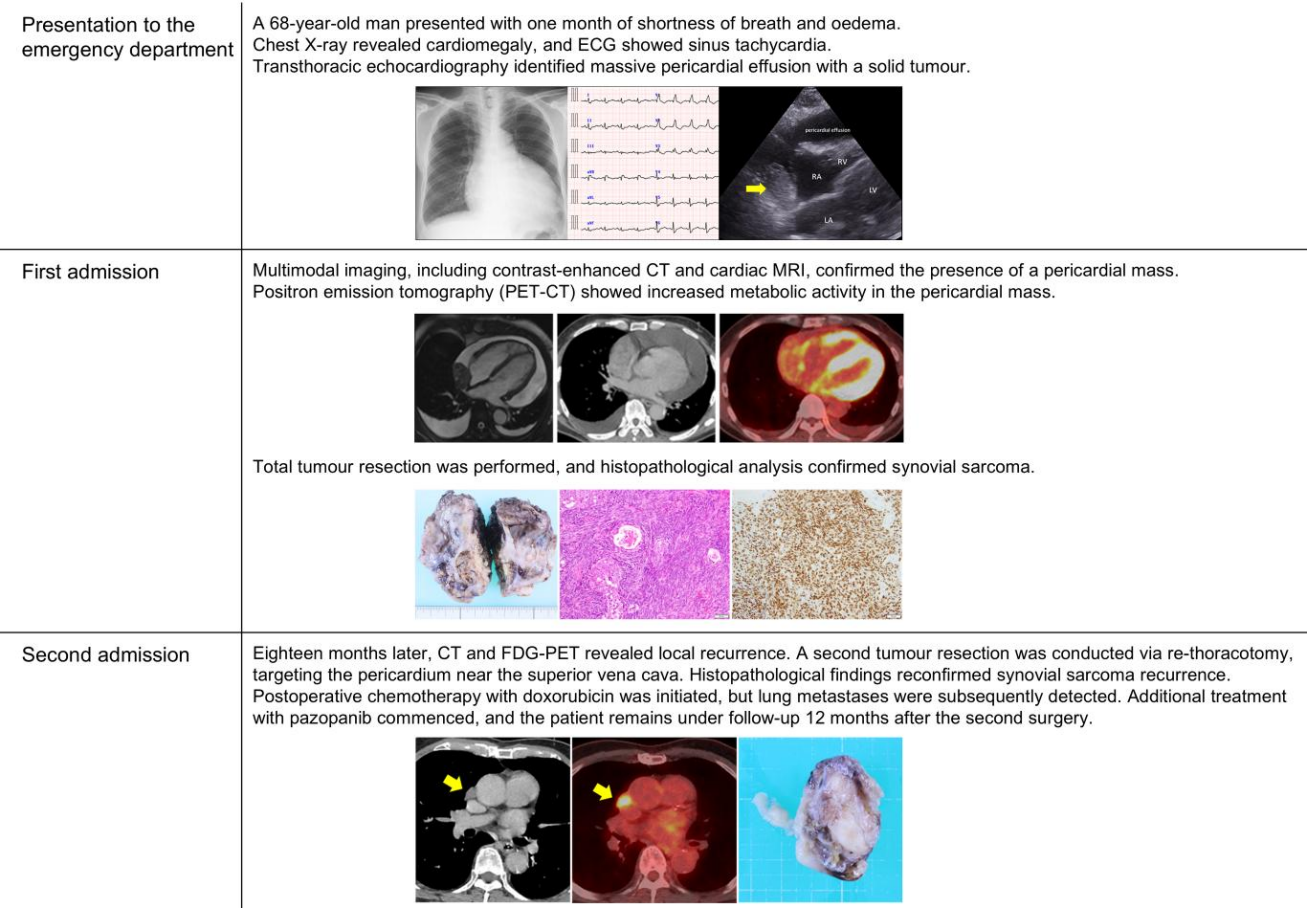


## Case presentation

A 68-year-old man presented with 1 month of shortness of breath and no significant medical history. Upon admission, his vital signs were as follows: blood pressure, 159/78 mmHg; pulse rate, 120 beats/min; respiratory rate, 18 breaths/min; temperature, 36.9°C; and oxygen saturation, 98%. Jugular vein distension was absent; however, he exhibited oedema in the eyelids and lower extremities.

**Figure 1:**
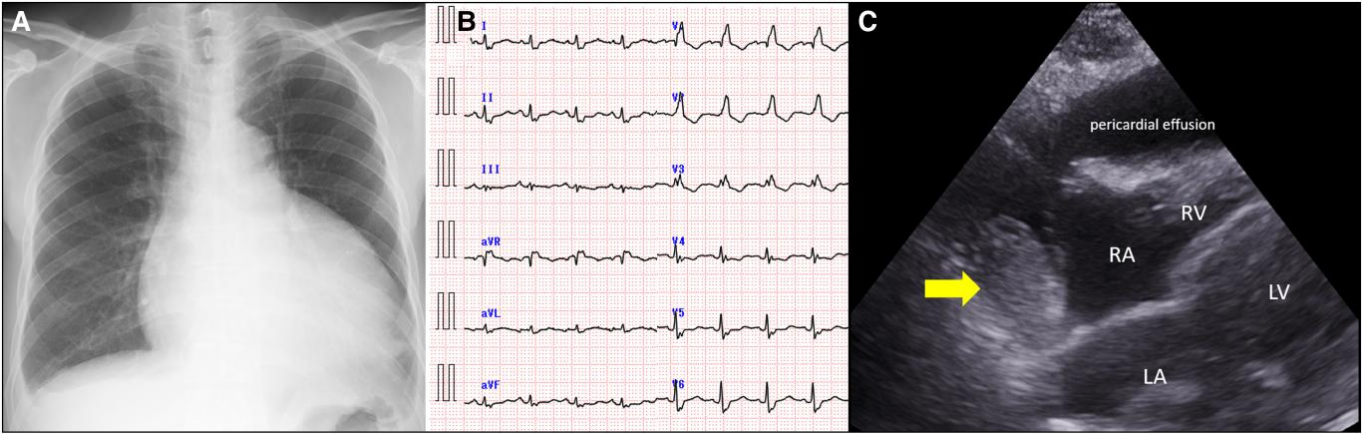
(*A*) A chest X-ray showed cardiomegaly and (*B*) electrocardiography showed sinus tachycardia, and (*C*) the transthoracic echocardiogram showed a large pericardial effusion and a solid tumour bordering the right atrium, indicated by a arrow.

Chest radiography demonstrated cardiomegaly with a cardiothoracic ratio of 66% (*Figure 1A*). Electrocardiography showed sinus tachycardia with a low-voltage QRS complex (*Figure 1B*). Transthoracic echocardiography (TTE) revealed a large pericardial effusion, right ventricular collapse, and a solid tumour with irregular borders in the right atrium (*Figure 1C*; Supplementary material online, *Video S1*).

**Figure 2:**
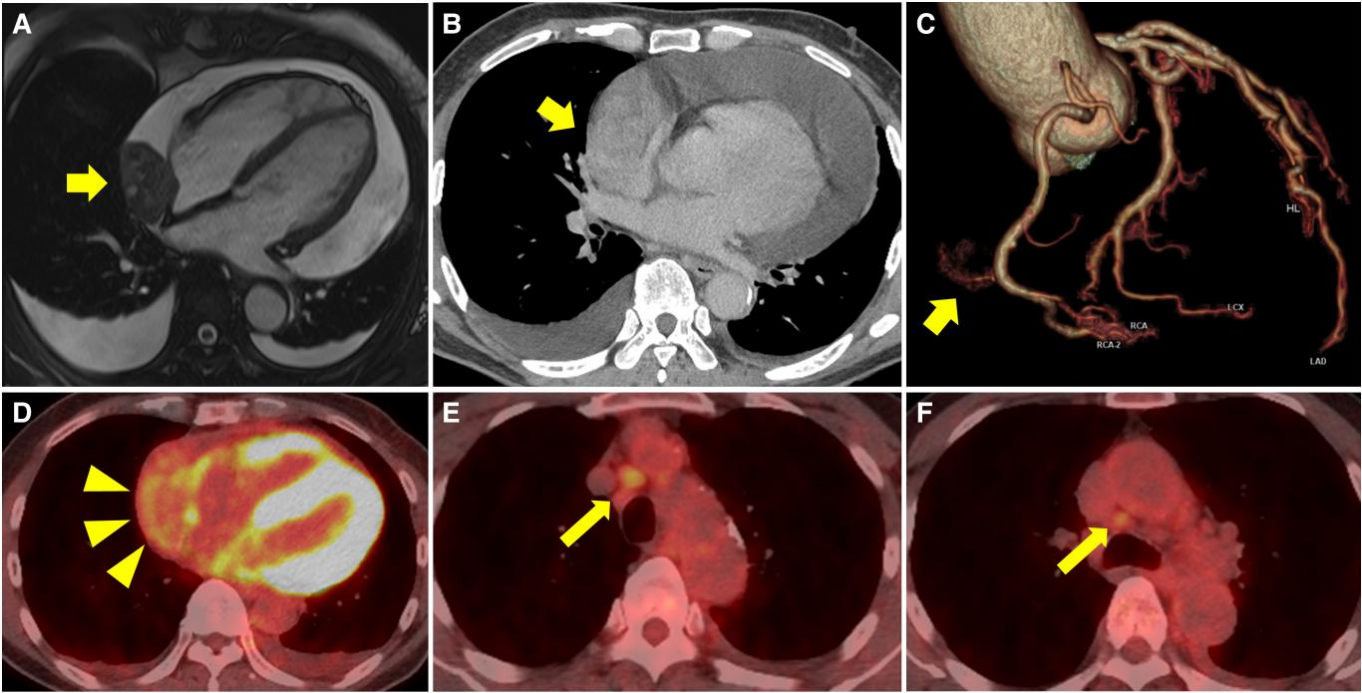
(*A* and *B*) Contrast-enhanced computed tomography (CT) and cardiac magnetic resonance imaging showed the mass, indicated by arrows and (*C*) coronary CT showed small arteries supplying the mass, also marked with a arrow, originating from the right coronary artery. (*D*) Positron emission tomography-CT showed increased uptake on the pericardial mass, indicated by arrowheads and (E and *F*) mediastinal lymph nodes, highlighted by long arrows.

Contrast-enhanced computed tomography (CECT) detected pericardial thickening, partial tumour calcification of the tumour, and delayed contrast enhancement (*Figure 2A*). Cardiac magnetic resonance imaging (CMR) showed a large pericardial effusion and an oval-shaped mass. The mass showed the low signal intensity on T1-weighted images and the high signal intensity on T2-weighted images. It also demonstrated heterogeneous gadolinium enhancement and a low apparent diffusion coefficient (ADC) value of 1.2 × 10⁻³ mm²/s, derived from diffusion-weighted imaging (*Figure 2B*). Additionally, coronary CT identified small feeding arteries originating from the right coronary artery (*Figure 2C*).

The laboratory data were as follows: white blood cell count, 11.8 × 10^3^ (3.3–8.6 × 10^3^) cells/μL; haemoglobin level, 14.1 (11.8–17.0) g/dL; platelet count, 237 × 10^3^ (158–348 × 10^3^) cells/μL; total protein, 7.0 (6.6–8.1) g/L; lactate dehydrogenase, 278 (124–222) U/L; C-reactive protein concentration, 1.09 (0.00–0.14) mg/dL; NT-proBNP, 427 (≤125) pg/mL; and soluble interleukin-2 receptor level, 630 (122–496) U/mL. Tumour markers, including carcinoembryonic antigen, carbohydrate antigen 19–9, cancer antigen 15–3, squamous cell carcinoma antigen, cytokeratin 19 fragment, cancer antigen 125, neuron-specific enolase, pro-gastrin-releasing peptide, and soluble interleukin-2 receptor, were not significantly elevated. Pericardial drainage yielded 500 mL of clear yellow serous fluid. Pericardial fluid analysis showed a protein level of 4.3 g/dL and lactate dehydrogenase of 224 U/L, suggestive of an exudate. No malignant cells were detected.

Positron emission-computed tomography (PET-CT) revealed increased uptake of fluorine-18-fluorodeoxyglucose in the pericardial mass [maximum standardised uptake value (SUVmax) 5.4] and in mediastinal lymph nodes (SUV max 2.8–3.3).

**Figure 3:**
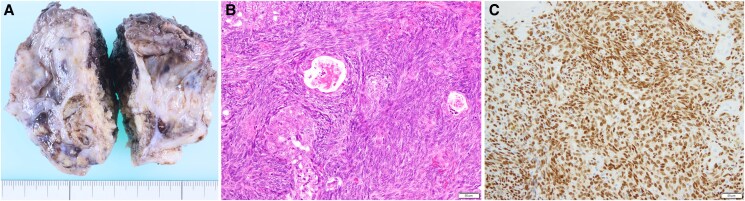
(*A*) White and substantial tumour (8.0 × 7.0 × 2.5 cm). (*B*) Histopathological examination of the tumour tissue samples showing proliferation of highly atypical spindle-shaped cells [haematoxylin and eosin (H&E); original magnification: × 200], (*C*) Immunohistochemistry: SS18-SSX staining was diffusely strongly positive (original magnification: × 200).

Thoracoscopic tumour biopsy revealed highly atypical spindle-shaped cells, prompting open total tumour excision and mediastinal lymph node dissection via median sternotomy. Pathological examination confirmed synovial sarcoma, which was diffusely positive for SS18-SSX, a specific fusion oncogene associated with the disease (*Figure 3 A–C*). No malignant tumours or metastases were observed in the lymph nodes; hence, no adjuvant therapy was administered.

**Figure 4:**
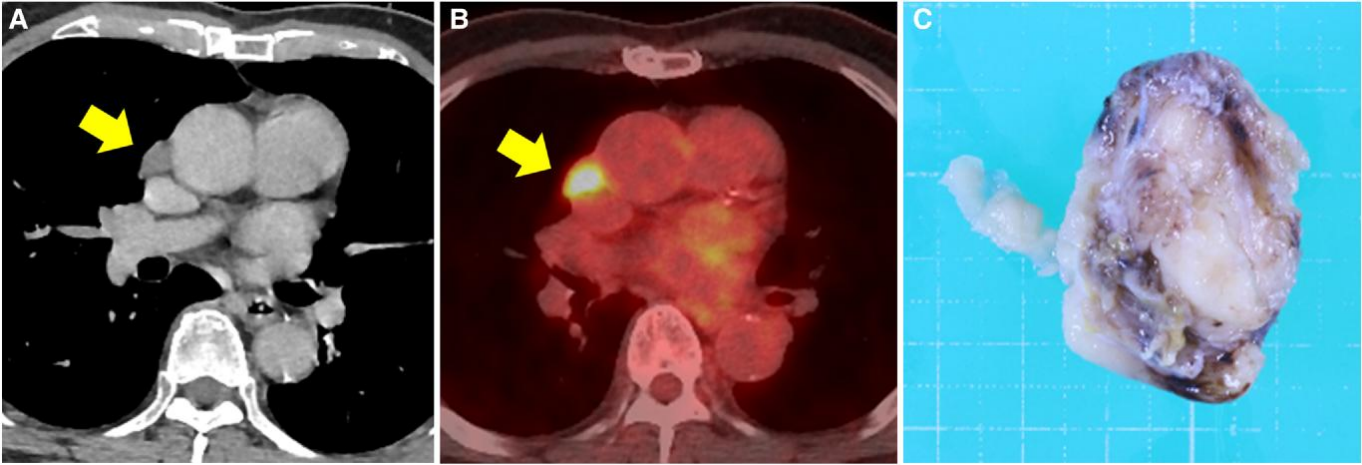
(*A* and *B*) Contrast-enhanced computed tomography (CT) and positron emission tomography-CT showed the mass, indicated by arrows. (*C*) The resected tumour (4.0 × 2.5 × 2.5 cm) was diagnosed the recurrence of synovial sarcoma by histopathological examination.

Following discharge, the patient was monitored using CECT and TTE every 3 months. Eighteen months post-surgery, CECT identified a mass between the right atrium and the superior vena cava (*Figure 4A*). PET-CT showed increased fluorine-18-fluorodeoxyglucose uptake in the mass (SUVmax 6.3) (*Figure 4B*) with evidence of metastasis, indicating local recurrence. The mass was surgically resected from the pericardium near the superior vena cava via resternotomy, while histopathology confirmed synovial sarcoma recurrence. Although postoperative chemotherapy with doxorubicin was administered, lung metastases were detected. Additional treatment with pazopanib was initiated 12 months after the second surgery.

## Discussion

This report presents a case of recurrent PSS identified using CECT 18 months after complete resection. Pericardial tumours are rare, with an incidence rate of <0.001% according to autopsy studies. Synovial sarcoma is a mesenchymal neoplasm accounting for 5–10% of soft tissue sarcomas. It is characterized by the t(X;18)(p11.2;q11.2) translocation, which results in the SS18-SSX fusion oncogene. This fusion gene, detected in >95% of cases, is the diagnostic gold standard.^3^ Histologically, synovial sarcomas can present in either a monophasic or biphasic pattern, comprising epithelial and/or spindle cell components.

The clinical presentation of PSS is atypical, ranging from asymptomatic to pericardial effusion with cardiac tamponade, dyspnoea, fever, weight loss, and embolic events.^1^ Owing to its nonspecific symptoms, the diagnosis and management of PSS rely on multimodal imaging. Echocardiography is the most used imaging modality. It can assess the location, size, shape, and mobility of masses as well as their relationships with adjacent structures and their haemodynamic impact.

PSS on echocardiography usually presents as an irregular, lobulated, inhomogeneous, hypoechoic mass, mostly accompanied by pericardial effusion and thickening, usually with a broad base, and only 24.1% of tumours are pedicled.^3^ A previous report suggests that the diagnostic value of echocardiography may be limited when a pericardial tumour presents simultaneously with massive pericardial effusion.^4^ Certain areas, such as the right heart system, may be obscured depending on the heart's morphology and the patient's physique. CT and CMR can visualize these blind spots, allowing for a more detailed assessment of the exact location, size, and other characteristics of the masses, alongside the information provided by echocardiography. CT scanning can provide detailed information about PSS features and its relationship with surrounding structures; assess the involvement of the mediastinum, lung, and pleura; and identify lymph nodes and distant metastases. On CT images, PSS typically appears as a mass with similar or slightly higher density, with either well-defined or indistinct boundaries. CMR is a superior non-invasive diagnostic tool offering high-resolution imaging for detailed characterization of PSS. It provides critical information on cardiac and pericardial structures, including tumour invasion into the myocardium. ADC values, which reflect restricted water movement in high-cell-density tumours, are typically decreased.^5^ PET-CT provides objective information on tumour metabolism, including glucose uptake, and is effective for the detection of metastasis to other organs. The SUV reflects tumour biology and aggressiveness, with a higher value indicating metabolic activity. The SUV values have been shown to correlate with tumour cellularity and mitotic rate in sarcomas.^6^ This suggests that PET-CT aids in differentiating tumours from thrombi, as the latter are typically metabolically 18F-fluorodeoxyglucose (FDG)-negative. In this case, pericardial effusion was observed on TTE. CT and magnetic resonance imaging (MRI) were used to identify the location of the mass, with MRI confirming high cellular density. Additionally, during the recurrence, CT detected a mass in the right atrium that was not visible on TTE. PET-CT showed a consistently high SUV max (5.4 at diagnosis, 6.3 at recurrence), confirming the metabolically active nature of the tumour differentiated from thrombi. This case highlighted the importance of multimodal evaluation for the diagnosis, treatment, and follow-up of cardiac synovial sarcomas.

Pericardial metastasis is common among cancer patients, with a prevalence of 10–20%.^7^ Pericardiocentesis can provide cytopathologic samples and relieve haemodynamic compromise. However, cytological results are often negative. In this case, while pericardiocentesis improved haemodynamics, no malignant cells were detected, which limited its role in establishing a definitive diagnosis. Adjunctive chemotherapy and radiotherapy may further enhance tumour regression and prolong survival, even when complete resection is achieved.^8^ Anthracycline-based chemotherapy has been significantly associated with improved overall survival in the general population and non-metastatic patients.^9^ A case series of 13 patients with synovial sarcoma treated with high-dose ifosfamide showed a response in all patients, with four achieving clinical remission. Combining doxorubicin with ifosfamide may lead to a higher response rate.^10^ Pazopanib efficacy in patients with soft tissue sarcoma who previously received chemotherapy with doxorubicin or ifosfamide was demonstrated in the PALETTE study,^11^ while its efficacy and safety were confirmed in a cohort.^12^ Radiotherapy, primarily used in patients with incomplete resection or unresectable disease, is associated with improved overall survival; however; further investigation is needed.^13^ Furthermore, evidence supporting the use of intrapericardial chemotherapy for pericardial synovial sarcoma is limited. While some studies have demonstrated its efficacy in malignant pericardial effusion related to lung cancer,^14^ its potential role in synovial sarcoma remains uncertain. In this case, the absence of malignant cells in the pericardial effusion precluded an evaluation of this treatment's applicability. In this case, complete resection was achieved because clear margins were obtained, and the resection margins were negative. However, 18 months after the initial surgery, recurrence was observed, while a second tumour resection was performed. Despite postoperative chemotherapy with doxorubicin, lung metastases were detected. Therefore, additional treatment with pazopanib was initiated. Given the clinical course, postoperative chemotherapy with pazopanib may be considered for patients with recurrent synovial sarcoma. Furthermore, considering the high risk of recurrence, pazopanib could have been considered as an adjuvant therapy following the initial complete resection, depending on patient-specific risk factors and clinical judgement.

## Conclusion

PSS is a rare and aggressive tumour with a high risk of recurrence even after complete surgical resection. This case highlights the importance of regular imaging surveillance, including multimodal approaches, for early detection of recurrence. Additionally, adjuvant chemotherapy may be effective, even after complete tumour resection, reducing the risk of recurrence.

## Lead author biography


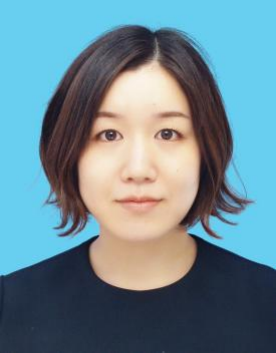


Kanna Nakamura was born in Osaka, Japan, in 1994. She received an MD degree from Osaka Medical and Pharmaceutical University, Osaka, Japan, in 2019. Her main areas of interest are echocardiography, cardiac magnetic resonance imaging, heart failure, and cardio-oncology.

## Supplementary Material

ytaf132_Supplementary_Data

## Data Availability

The data underlying this article will be shared on reasonable request to the corresponding author.

## Notes

**Consent:** The authors confirm that written consent for the submission and publication of this case report, including image(s) and associated text, has been obtained from the patient in accordance with COPE guidance.

**Funding:** None declared.
